# Thrombus Imaging Features for Anterior Circulation Stroke: Their Impact on CTP Parameters and Natural Evolution of Infarct Progression

**DOI:** 10.3390/jpm15100464

**Published:** 2025-10-01

**Authors:** Bruna G. Dutra, Heitor C. B. R. Alves, Vivian Gagliardi, Rubens J. Gagliardi, Felipe T. Pacheco, Antonio C. M. Maia, Antônio J. da Rocha

**Affiliations:** 1 Department of Radiology and Oncology, Universidade de São Paulo, São Paulo 05508-060, SP, Brazil; heitor.alves@hc.fm.usp.br; 2 Fleury Group, São Paulo 04344-070, SP, Brazil; antonio.maia@fleury.com.br; 3 Irmandade Santa Casa de Misericórdia de São Paulo, São Paulo 01221-020, SP, Brazil; viviandbg@gmail.com (V.G.); rubensjg@apm.org.br (R.J.G.); felipe.pacheco.ext@dasa.com.br (F.T.P.); a.rocha@uol.com.br (A.J.d.R.); 4 DASA Group, São Paulo 06455-010, SP, Brazil

**Keywords:** acute ischemic stroke (AIS), clot burden score (CBS), cerebral blood volume (CBV), computed tomography perfusion (CTP), large vessel occlusion (LVO), time-to-maximum value (tmax)

## Abstract

**Background/Objectives**: The relationship between thrombus imaging features and the natural evolution of stroke remains poorly defined. We aimed to investigate the associations between thrombus characteristics on CT and perfusion parameters, as well as subsequent infarct progression, in untreated patients experiencing an anterior circulation acute ischemic stroke (AIS). **Methods**: This retrospective analysis enrolled 81 untreated patients with AIS who underwent baseline non-contrast CT (NCCT), CT angiography (CTA), CT perfusion (CTP), and a follow-up NCCT. We evaluated the thrombus length, location, and clot burden score (CBS). CTP parameters included the ischemic core, hypoperfused tissue, and penumbra volumes. Infarct growth was the difference between the final infarct volume on a follow-up NCCT and the initial core volume on CTP. Univariate and multivariate regression models were performed. **Results**: Higher CBS values and shorter thrombi are associated with a reduced ischemic core (coefficients B of −3.9 and 0.88, *p* < 0.01), diminished hypoperfused tissue (coefficients B of −12.2 and 2.87, *p* < 0.001), and smaller penumbra volume (coefficients B of −7.9 and 1.99, *p* < 0.001). More distal occlusions were associated with smaller perfusion deficits. Importantly, a higher CBS and more distal thrombus location were significantly associated with a smaller final infarct volume and infarct growth volume. **Conclusions**: In untreated AIS patients, a lower thrombus burden (higher CBS, shorter length, distal location) is associated with more favorable baseline perfusion parameters and predicts a slower, less severe natural evolution of AIS. These findings underscore the prognostic value of baseline thrombus characteristics in determining the intrinsic course of a stroke.

## 1. Introduction

Acute ischemic stroke (AIS) is a leading cause of morbidity and mortality worldwide. It remains a critical medical emergency, demanding precise and timely interventions to prevent the progression of irreversible infarcted tissue [1].

The vessel occlusion triggers the intricate landscape of brain ischemia and infarct progression. This landscape is influenced by a multitude of factors, including collateral circulation, thrombus imaging characteristics, time from stroke onset, time to treatment, and reperfusion therapies [2,3,4,5]. Previous studies have reported that proximal thrombi are associated with a larger ischemic core and penumbra volumes [6,7]. Despite this knowledge, a paucity of data remains concerning the nuanced relationships between thrombus imaging characteristics and perfusion parameters, as well as infarct growth, especially in untreated patients [8].

A key aspect in the treatment of AIS lies in determining the extent of ischemic injury from the potentially salvageable ischemic penumbra. This distinction is crucial for guiding appropriate reperfusion strategies [9]. Within this diagnostic context, computed tomography perfusion (CTP) has emerged as a possible imaging modality for identifying ischemic and salvageable tissues, particularly within the extended time window of AIS [2,9,10,11]. Perfusion imaging, while informative, provides a snapshot in time, capturing perfusion parameters only at the time of acquisition [12,13]. It does not fully capture the dynamic individual progression of at-risk penumbral tissue towards irreversible infarction, which can vary significantly among patients even with similar initial perfusion maps.

Our objective was to investigate the characteristics of thrombus imaging and their association with the natural evolution of stroke. Specifically, our goal is to clarify the relationship between these imaging features and initial perfusion deficits, as well as their direct connection to future infarct progression in untreated AIS patients. A comprehensive understanding of these natural interrelationships is essential to acknowledge the significance and importance of thrombus imaging features in stroke evolution, before their broader application as therapeutic targets in clinical practice. Such models could enable clinicians to estimate individual perfusional patterns and predict infarct evolution based on more readily accessible thrombus imaging characteristics. This understanding is particularly significant for enhancing individualized stroke care in settings with limited access to advanced imaging modalities, especially in low-resource settings in developing countries, thereby fostering more equitable and individually optimized stroke management.

## 2. Materials and Methods

### 2.1. Study Design and Patient Selection

We performed a retrospective cohort study on all patients with suspected AIS admitted to Hospital Santa Casa de Misericórdia de São Paulo, Brazil, from July 2011 to December 2018. The local Ethics Committee approved the study (protocol number: 62124816.7.0000.5479). Written consent was obtained from all participants before examinations were performed.

The following inclusion criteria were used: patients with an anterior circulation AIS; aged 18 years or older; a stroke onset time of 8 h or less; and patients who underwent thin-slice (≤2.5 mm) baseline non-contrast computed tomography (NCCT), computed tomography angiography (CTA), and computed tomography perfusion (CTP). Exclusion criteria were the following: unavailable baseline clinical data; patients treated with intravenous thrombolysis and/or endovascular treatment; incomplete CT images; scans with motion artifacts, excess noise, or poor contrast opacification on CTA or CTP; anterior cerebral artery or cervical internal carotid artery occlusions; no depicted intracranial occlusion; bilateral AIS or evidence of a hemorrhage on baseline or follow-up scans.

### 2.2. Imaging Protocol and Analyses

All patients underwent thin-slice baseline NCCT, CTA, and CTP acquired within 10 min on a 64-channel multi-slice computed tomography (Brilliance CT 64 Channel, Philips Medical, Eindhoven, The Netherlands). The CTP imaging covered a *z*-axis range of 4 cm. We optimized the field of view of CTP slices to include the greater suspected ischemic area depicted on NCCT before CTP acquisition. When no suspected ischemic area was depicted on CTA, the field of view was positioned on the level of the basal ganglia. We typically performed a follow-up NCCT scan at 24 to 48 h from stroke onset. However, a few patients had a late follow-up scan performed up to 5 days after the baseline imaging. Imaging analyses were performed by two stroke research neuro-radiologists with over 8 years of experience (B.D. and H.A.), using baseline NCCT with the aid of the co-registered baseline CTA. Readers were blinded to all patient clinical data. The CTP parameters (H.A.), thrombus length (B.D.), and final infarct volume (B.D.) were measured by one radiologist. CBS and thrombus location were assessed independently by two radiologists; for cases in which the assessments differed, a consensus was reached. When assessing NCCT and CTA imaging parameters, the readers were blinded to the CTP results to prevent bias.

**Figure 1:**
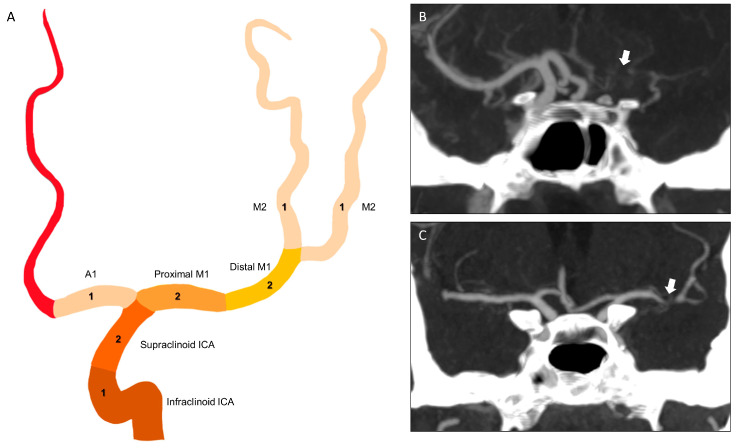
(**A**) Schematic illustration of the clot burden score (CBS). Points are subtracted (indicated by the number inside the arteries) for each arterial segment occluded by the thrombus. (**B**,**C**) CTA coronal views demonstrate different measurements of CBS. (**B**) The first patient presents with a thrombus occluding the infra and supraclinoid ICA segments of the left ACI (arrow), as well as segments A1, proximal M1, distal M1, and the two branches of homolateral M2, resulting in a CBS of 0. (**C**) Another patient has a thrombus in the distal M1 segment of the left middle cerebral artery (arrow), corresponding to a CBS of 8.

The thrombus was assessed using the following CT thrombus imaging characteristics: location, length, and clot burden score (CBS). The thrombus location was based on the most proximal vascular segment occluded on baseline CTA and was classified as an intracranial internal carotid artery (ICA) (intracranial ICA: included in the lacerum, cavernous and ophthalmic segments of ICA), terminal ICA (ICA-t), or part of the middle cerebral artery segments (proximal M1, distal M1, M2, M3, and M4). Thrombus length was estimated by the extent of the occluded arterial segment depicted on CTA, using multiplanar reformations. With the aid of co-registered NCCT, we used the hyperdense artery sign to estimate the thrombus length when the proximal or distal part of the thrombus could not be identified on CTA. Whenever the thrombi extended into two arterial branches, length measurements were performed in the branch that resulted in the longest thrombus. The CBS was assessed on CTA, according to Puetz et al. [2]. It is a semiquantitative method that combines features of thrombus length and location (Figure 1).

The CTP analyses were performed using Olea Sphere^®^ software, version 3.0 (Olea Medical Solutions, La Ciotat, France), and the Bayesian deconvolution method. We evaluated the following CTP parameters: core infarct volume, hypoperfused tissue volume, and penumbra volume. Core infarct and hypoperfused volumes were assessed using default settings. The core infarct was defined as the region with a relative cerebral blood flow less than 40% and a time-to-maximum value greater than 2 s compared to the contralateral side [14]. Hypoperfused tissue was calculated as a time-to-maximum value exceeding 6 s [14]. Penumbra volume was defined as the mismatch volume between the hypoperfusion and core volume [11].

We also evaluated the early infarct growth rate, estimated by dividing the ischemic core on CTP by the time from symptom onset to baseline CTP imaging. The final infarct volume was assessed in the 24–48 h NCCT using the ABC/2 method [15]. Infarct growth volume was defined as the difference between the final infarct volume on the follow-up NCCT scan and the ischemic core volume depicted on CTP.

### 2.3. Statistical Methods

We describe the baseline clinical and imaging characteristics of our study populations, using medians and interquartile ranges (IQRs) for continuous variables, and frequencies and percentages for categorical variables. The interrater agreement for CBS and thrombus location was assessed between the two readers using weighted Cohen’s kappa statistics.

Univariable and multivariable linear and binary regression models were used to assess the associations between thrombus imaging characteristics (length, CBS, and location) and the ischemic core, hypoperfused tissue, penumbra volume, final infarct volume, infarct growth volume, and early infarct growth rate. We believe that collaterals are part of the causal pathway between the thrombus and outcomes as follows: distal and short thrombi likely correlate with more patent Willisian routes, resulting in improved pial collaterals, enhanced CTP parameters, reduced infarct growth, and a decrease in the final infarct volume [4,16,17,18,19]. However, to prevent the potential omitted variable bias, we incorporated the collateral score, age, and sex into all the multivariate models to evaluate the robustness of our findings. We added time from stroke onset to baseline imaging as part of the adjustments for the analyses with the core, hypoperfused tissue, penumbra volumes, final infarct volume, and infarct growth volume as outcome measurements.

All statistics were obtained using R (R Foundation for Statistical Computing, Vienna, Austria, version 4.3.1).

## 3. Results

### 3.1. Subjects’ Characteristics

**Figure 2:**
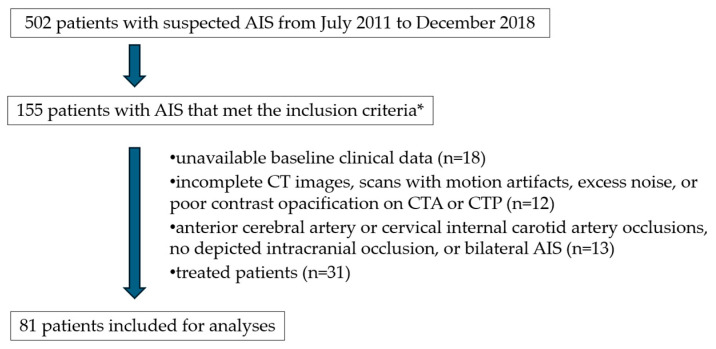
Flowchart for patient selection. AIS: acute ischemic stroke; CT: computed tomography; CTA: computed tomography angiography; CTP: computed tomography perfusion; n: number * Inclusion criteria: patients with an anterior circulation AIS; age ≥ 18 years; stroke onset ≤ 8 h; and patients who underwent thin-slice (≤2.5 mm) baseline NCCT, CTA, CTP.

The flowchart for patient selection is detailed in Figure 2. Our final study population consisted of 81 patients with untreated AIS.

The patients’ baseline characteristics are listed in Table 1. Interrater agreements for CBS and thrombus location, measured by Cohen’s kappa, were 0.81 and 0.85, respectively.

The median age was 62 years (IQR, 55–77); 55.6% (n = 45) were male; and the median baseline National Institutes of Health Stroke Scale score was 11.5 (IQR, 6–16). The median CBS value was 6 (IQR of 5 to 9), and the median thrombus length was 12.6 mm (IQR, 7.5–18). Intracranial ICA thrombus was the most common location (24.7%), followed by distal M1 thrombus (18.5%). The median values for the infarct core volume and hypoperfused volume were 7.6 mL (IQR, 1.6–25) and 74.6 mL (IQR, 27.2–123), respectively.

Follow-up NCCT scans were performed in 54 patients (66.7%). The median values for final infarct volume and infarct growth volume were 50 mL (IQR, 22.7–154) and 116 mL (IQR, 32–252), respectively.

### 3.2. Outcome Analyses

A higher CBS and shorter thrombus length were significantly associated with a smaller ischemic core volume (coefficients B of −3.9 and 0.88, *p* < 0.01), smaller hypoperfused tissue volume (coefficients B of −12.2 and 2.87, *p* < 0.01), and smaller penumbra volume (coefficients B of −7.9 and 1.99, *p* < 0.01). Occlusions of M2 or more distal segments had a smaller ischemic core, hypoperfused tissue, and penumbra volume than ICA occlusions. Compared to ICA occlusions, M2 occlusions had an approximately 23 mL decrease in the ischemic core volume (coefficient B, −23.5; 95% CI, −39.3 to −7.7, *p* < 0.001), a 64 mL decrease in hypoperfused tissue volume (coefficient B, −64.3; 95% CI, −94.5 to −34.1), and a 39 mL decrease in penumbra volume (coefficient B, −38.8; 95% CI, −64.6 to −13.1). Detailed results are provided in Table 2.

A higher CBS was associated with a lower early infarct growth rate (coefficient B = −2.3; 95% CI, −4.2 to −0.5; *p* = 0.01), smaller final infarct volume, and reduced infarct growth volume. Similarly, more distally located thrombi were associated with smaller final infarct and growth volumes. The magnitude of this effect was substantial; compared to ICA occlusions, M2 occlusions were statistically significant and associated with an approximately 198 mL decrease in final infarct volume and a 197 mL decrease in infarct growth volume. Thrombus length and location did not show a significant association with the rate of early infarct growth. Additionally, thrombus length did not have a significant impact on the final infarct volume or infarct growth volume. Table 3 provides a detailed summary of the results.

**Figure 3:**
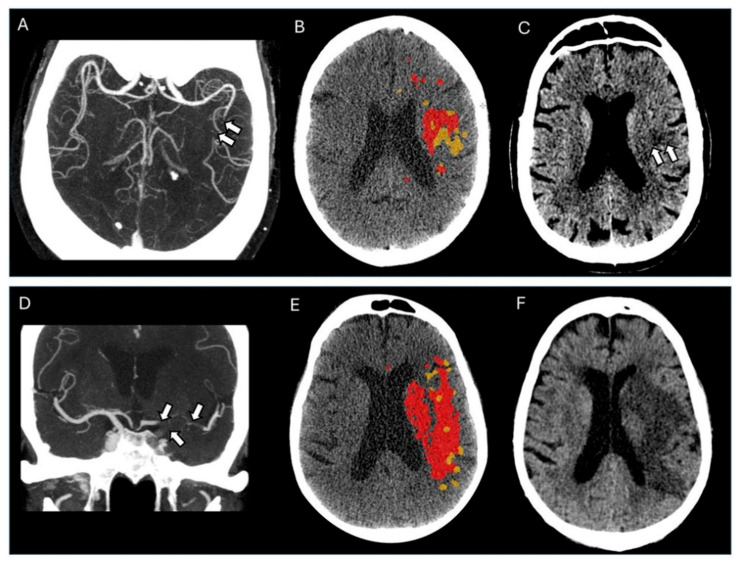
(**A**–**C**) A patient with a distal thrombus. (**A**) An axial CTA view shows an occlusion in the M2 segment of the left middle cerebral artery (arrows), resulting in a CBS of 9. The thrombus length was 12.5 mm. (**B**) CTP demonstrates a small core volume (red) and a small hypoperfused volume (yellow). (**C**) An NCCT at 1-day follow-up shows a small final infarct volume (arrows). (**D**–**F**) A patient with a proximally located thrombus. (**D**) A CTA coronal view showing a thrombus in the left ICA-t segment, extending to the A1 and proximal and distal M1 segments (arrows). The CBS was 3, and the thrombus length was 23.6 mm. (**E**) CTP shows a large core volume (red) and hypoperfused volume (yellow). (**F**) A 48-h follow-up NCCT demonstrates a large final infarct volume.

Examples of the association between thrombus imaging features and perfusion parameters alongside final infarct volume are illustrated in Figure 3.

**Figure 4:**
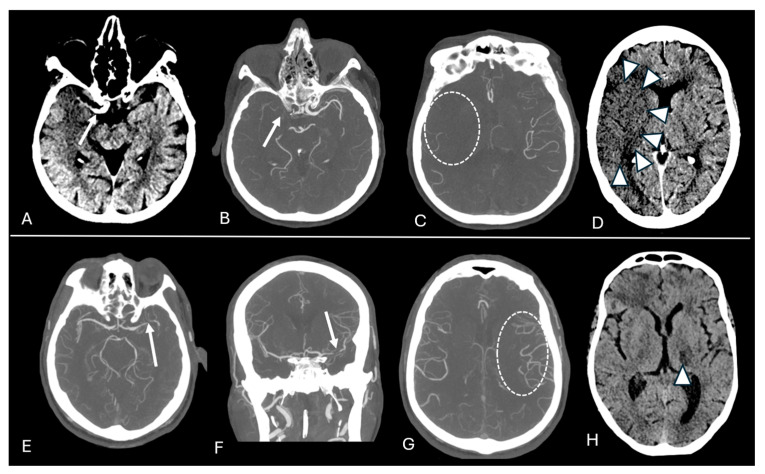
(**A**–**D**) A patient with a higher thrombus burden. (**A**) Axial NCCT and (**B**,**C**) CTA views show an occlusion in the right intracranial ICA segment (arrows). A poor collateral score is depicted (dashed circle). (**D**) An NCCT at 2-day follow-up shows a large final infarct volume in the right MCA territory (arrowheads). (**E**–**H**) A patient with a lower thrombus burden. (**E**–**G**) CTA views show a thrombus in the left distal M1 segment (arrows) and a good collateral score (dashed circle). (**H**) An NCCT at 1-day follow-up shows a small core volume (arrowhead) in the left thalamus.

After including the confounders in the multivariate analyses, most of the primary associations remained significant. However, these adjustments rendered the associations between ischemic core volume and thrombus length, as well as M3/M4 occlusions, statistically non-significant. We observed that the significant relationship between CBS and early infarct growth rate was nullified after adjusting for collateral status, sex, and age. This finding suggests that these covariates might be mediating factors in these specific interactions. Multivariate analyses are listed in Appendix A (Table A1 and Table A2). Examples of the association between thrombus imaging features and perfusion parameters, as well as final infarct volume, are illustrated in Figure 4.

## 4. Discussion

We assessed the association between thrombus imaging characteristics and CTP parameters alongside infarct evolution in untreated AIS patients. We found that a greater CBS and shorter thrombi were correlated with a reduced ischemic core, diminished hypoperfused tissue, and smaller penumbra volume. Furthermore, a higher CBS was found to be associated with a reduced early infarct growth rate, decreased final infarct volume, and smaller infarct growth volume. Thrombi located more distally were associated with a reduced ischemic core, diminished hypoperfused tissue, smaller penumbra volume, and smaller final infarct volume and infarct growth volume. Specifically, thrombi positioned at the M2 segment or more distally were associated with smaller core, hypoperfused tissue, and penumbra volumes. After adjusting for collateral status, age, and sex, the previously significant associations of thrombus length and M3/M4 location with the ischemic core volume were rendered non-significant. The association between CBS and the early infarct growth rate was also nullified after the adjustments.

Our results directly support the mechanistic concept that a larger, proximal thrombus creates a more profound and widespread hemodynamic failure, overwhelming the capacity of collateral pathways and establishing a larger territory of at-risk tissue from onset [6,7,20].

The existing literature highlights a need for more data on the associations between thrombus imaging characteristics and CTP parameters in AIS, particularly in untreated patients. We were unable to identify previous studies that have systematically evaluated the association between thrombus imaging features and perfusion deficits exclusively in a cohort of untreated AIS patients. Sillanpäa et al. [6] observed a significantly shorter mean transit time (MTT) and penumbra defects associated with thrombi located more distally in stroke patients receiving intravenous thrombolytic therapy. Notably, M2 and M3 thrombi demonstrated markedly reduced cerebral blood volume (CBV) defects compared to ICA thrombi. Although Sillanpäa et al. [6] employed CTP ASPECTS mismatch to estimate perfusion defect size and MTT to assess hypoperfused tissue, their findings closely resonate with the results of our study. Moreover, Gasparotti et al. [21] also showed that ICA-t occlusions have a larger infarct core than MCA occlusions in patients treated with intra-arterial thrombolysis. Concurrently, Gawlitza et al. [20] established a correlation between an increased distance from the carotid terminus to the occlusion site, resulting in a lower mismatch volume and reduced likelihood of target mismatch. Gawlitza et al. [20] also evaluated AIS patients treated with intravenous thrombolysis. A possible explanation for the reduced likelihood of target mismatch in distal thrombus locations is the typically smaller core and hypoperfused volumes associated with these thrombi, failing to meet target mismatch criteria. In the context of thrombus imaging features, our findings align with previous research, which found a correlation between thrombus burden and smaller core and penumbra volumes, resulting in a smaller final infarct volume [2,7,22,23,24,25]. A higher infarct growth rate was also observed with more proximal thrombi, although the literature addressing this relationship is limited [1,24].

The intricate association between thrombus imaging characteristics and infarct progression in AIS could be attributed to the pathophysiological mechanisms of vascular occlusion and subsequent tissue hypoperfusion. A higher thrombus burden (proximally located, higher length, and lower CBS) is correlated with a more extensive vascular compromise, leading to a continuum of ischemic injury ranging from reversible ischemic penumbra to irreversible infarct core. The ischemic penumbra is susceptible to infarct expansion, particularly when collateral blood flow is insufficient to maintain cellular function in the context of a high thrombus burden. This insufficiency results in rapid depletion of neuronal energetic reserves and accelerates the conversion of penumbral tissue into a necrotic infarct [7,26,27,28].

Our multivariate analysis highlights the potential mediating role of collateral circulation, an effect observed in a minority of the relationships we investigated. After adjustments, the associations between some thrombus features (length and M3/M4 location) and ischemic core volume, as well as the relationship between CBS and the rate of early infarct growth, lost statistical significance. This finding suggests that collateral flow may be a crucial factor in the causal pathway linking thrombus characteristics to tissue outcomes, a mechanism well-supported by prior studies [4,7,17,27,28,29,30,31]. Our findings in an untreated cohort provide direct evidence for this model: a smaller, more distal thrombus (i.e., a higher CBS) likely permits more effective collateral recruitment, which, in turn, protects brain tissue and mitigates the extent of the core infarction [31,32].

The clinical implications of this research are particularly relevant for prognostication and guiding management in resource-limited settings [33]. Where advanced imaging, such as CTP, is not readily available, a simple assessment of thrombus characteristics on CTA can provide invaluable prognostic information about the most likely natural course of the stroke. Identifying a patient with a low-burden, distal thrombus may suggest a slower progression of AIS. In contrast, a high-burden, proximal thrombus signals a high likelihood of rapid and extensive infarction, which is a concept that can inform clinical decision-making and patient counseling.

Our analyses have several limitations, including their retrospective nature and a modest sample size. First, incomplete clinical outcomes were found from medical records, particularly regarding the modified Rankin Scale at 90 days. Moreover, reperfusion status was not reported; this information was lacking in medical records, and there was an absence of a follow-up CTA scan. However, our primary objective was to investigate the pathophysiology of infarct progression using imaging endpoints, which provide a direct measure of biological evolution. Second, the data were collected between 2011 and 2018; however, we used the latest version of the software for CTP analysis to mitigate this limitation. Third, the restricted brain coverage on CTP (4 cm) may underestimate the ischemic and hypoperfused volumes; however, we optimized the field of view of CTP slices in the greater suspected hypoattenuated area on NCCT before CTP acquisition. Fourth, the small sample size of M3 and M4 occlusions may impact the generalizability of our conclusions regarding more distally located occlusions. These findings warrant further investigation into larger cohorts.

## 5. Conclusions

Thrombus imaging characteristics are powerful determinants of the natural course of infarct evolution. A lower thrombus burden, as indicated by a higher CBS, shorter length, and more distal location, is associated with smaller perfusion deficits and, most importantly, reduced subsequent infarct growth. Our findings suggest that collateral circulation may mediate the minority of relationships between some thrombus imaging features and outcomes. Our work enhances our fundamental understanding of stroke pathophysiology. It highlights the significant prognostic value of baseline thrombus imaging, providing crucial insights that can inform patient management, especially in resource-constrained environments where access to advanced imaging, such as CTP, remains limited. Moreover, thrombus imaging could offer an invaluable, rapid first assessment to guide initial management and triage decisions, thereby democratizing elements of personalized stroke care. Thus, further studies with larger prospective cohorts are imperative to precisely elucidate the role of thrombus imaging features in influencing treatment decisions and ultimately improving patient outcomes.

## Figures and Tables

**Table 1:** Patients’ baseline characteristics included in our study.

	Total = 81 Patients
Clinical variables	
Age, median (IQR)	62 (55, 77)
Male sex, n (%)	45 (55.6)
Baseline NIHSS, median (IQR)	11.5 (6, 16)
Stroke ictus, min—median (IQR)	120 (60, 215)
Systolic blood pressure, mm Hg, median (IQR)	150 (130, 180)
Diastolic blood pressure, mm Hg—median (IQR)	90 (80, 100)
Baseline glucose, mg/dL—median (IQR)	130 (112, 171)
Medical history	
Current smoker	19 (25)
Hypertension	45 (67.1)
Diabetes	17 (22.4)
Arterial fibrillation	17 (22.4)
Dyslipidemia	6 (7.9)
Peripheral artery disease	4 (5.3)
Previous stroke	8 (10.5)
Previous myocardial infarction	6 (7.9)
Anticoagulation	4 (5.3)
Imaging variables	
ASPECTS, median (IQR)	7 (5, 8)
Occlusion location, n (%)	
ICA-i	5 (6.2)
ICA-t	15 (18.5)
Proximal M1	12 (14.8)
Distal M1	15 (18.5)
M2	19 (23.5)
M3	13 (16)
M4	2 (2.5)
Tandem occlusion, n (%)	4 (5.5)
CBS, median (IQR)	6 (5, 9)
Collateral score, n (%)	
0% filling of the occluded territory	12 (14.8)
0–50% filling of the occluded territory	21 (25.9)
50–100% filling of the occluded territory	25 (30.9)
100% filling of the occluded territory	23 (28.4)
Hyperdense artery sign, n (%)	48 (60)
Thrombus length—mm, median (IQR)	12.6 (7.5, 18)
CTP parameters	
Infarct core volume, mL—median (IQR)	7.6 (1.6, 25)
Hypoperfused tissue volume, mL—median (IQR)	74.6 (27.2, 123)
Penumbra volume, mL—median (IQR)	50.5 (21.7, 100)
Early infarct growth rate, mL/h—median (IQR)	39.7 (22.2, 151)
Follow-up imaging variables *	
Follow-up scan date, median (IQR)	2 (1, 4)
Final infarct volume, mL—median (IQR)	50 (22.7, 154)
Infarct growth volume, mL—median (IQR)	116 (32, 252)

ASPECTS, Alberta Stroke Program Early CT Score; ICA-i, intracranial segment of internal carotid artery; ICA-t, terminal segment of internal carotid artery; IQR, interquartile range; M1, segment M1 of the middle cerebral artery; M2, segment M2 of the middle cerebral artery; M3, segment M3 of the middle cerebral artery; M4, segment M4 of the middle cerebral artery; n = number. * Follow-up NCCT scans were performed on 54 patients.

**Table 2:** Associations of thrombus imaging characteristics with ischemic core volume, hypoperfused tissue volume, and penumbra volume.

	Ischemic Core Volume			Hypoperfused Tissue Volume			Penumbra Volume		
	β (95%CI)	R-Squared	*p*-Value	β (95%CI)	R-Squared	*p*-Value	β (95%CI)	R-Squared	*p*-Value
Clot Burden Score	−3.9 (−5.7 to −2.1)	0.18	<0.001	−12.2 (−16.1 to −8.4)	0.34	<0.001	−7.9 (−11.3 to −4.6)	0.22	<0.001
Thrombus length	0.88 (0.2 to 1.5)	0.08	<0.01	2.87 (1.6 to 4.1)	0.23	<0.001	1.99 (0.9 to 3.1)	0.17	<0.001
Thrombus location									
ICA	1 (Reference)			1 (Reference)			1 (Reference)		
Proximal M1	−6.3 (−23.5 to 10.7)	0.13	0.46	−7.7 (−40.3 to 24.8)	0.39	0.63	0.5 (−27.3 to 28.4)	0.31	0.96
Distal M1	−13.3 (−31.2 to 4.5)	0.13	0.14	−25.9 (−60.1 to 8.1)	0.39	0.13	−10.7 (−39.8 to 18.4)	0.31	0.47
M2	−23.5 (−39.3 to −7.7)	0.13	<0.001	−64.3 (−94.5 to −34.1)	0.39	<0.001	−38.8 (−64.6 to −13.1)	0.31	<0.001
M3/M4	−28.4 (−45.5 to −11.9)	0.13	<0.001	−102.4 (−135.1 to −69.8)	0.39	<0.001	−72.1 (−99.9 to −44.1)	0.31	<0.001

CI: confidence interval; ICA, internal carotid artery; M1, segment M1 of the middle cerebral artery; M2, segment M2 of the middle cerebral artery; M3, segment M3 of the middle cerebral artery; M4, segment M4 of the middle cerebral artery.

**Table 3:** Associations of thrombus imaging characteristics with early infarct growth rate, final infarct volume, and infarct growth volume.

	Early Infarct Growth Rate			Final Infarct Volume			Infarct Growth Volume		
	β (95% CI)	R-Squared	*p*-Value	β (95% CI)	R-Squared	*p*-Value	β (95% CI)	R-Squared	*p*-Value
Clot Burden Score	−2.3 (−4.2 to −0.5)	0.07	0.01	−22.8 (−33.4 to −12.3)	0.25	<0.001	−22.7 (−32.9 to −12.6)	0.27	<0.001
Thrombus length	0.38 (−0.26 to 1.02)	0.02	0.24	3.5 (−0.58 to 7.62)	0.05	0.06	3.1 (−0.78 to 7.03)	0.05	0.11
Thrombus location									
ICA	1 (Reference)			1 (Reference)			1 (Reference)		
Proximal M1	−0.53 (−17.6 to 16.5)	0.01	0.95	−134.5 (−233.5 to −45.5)	0.29	<0.001	−155.9 (−240.9 to −70.9)	0.33	<0.001
Distal M1	−4.4 (−22.3 to 13.6)	0.01	0.62	−105.4 (−199.7 to −10.9)	0.29	0.02	−109.4 (−197.1 to −21.8)	0.33	0.01
M2	−10.7 (−26.6 to 5.1)	0.01	0.18	−197.7 (−286.7 to −108.7)	0.29	<0.001	−196.9 (−279.6 to −114.1)	0.33	<0.001
M3/M4	−16.1 (−33.1 to −1.0)	0.01	0.06	−185.6 (−280.1 to −91.2)	0.29	<0.001	−182.9 (−270.5 to −95.4)	0.33	<0.001

CI: confidence interval; ICA, internal carotid artery; M1, segment M1 of the middle cerebral artery; M2, segment M2 of the middle cerebral artery; M3, segment M3 of the middle cerebral artery; M4, segment M4 of the middle cerebral artery.

## Data Availability

The data that support the findings of this study are available from the corresponding author upon reasonable request due to privacy and legal reasons.

## Abbreviations

The following abbreviations are used in this manuscript:

AIS	acute ischemic stroke
CBS	clot burden score
CBV	cerebral blood volume
CTA	computed tomography angiography
CTP	computed tomography perfusion
ICA	internal carotid artery
NCCT	non-contrast computed tomography
Tmax	time-to-maximum value

## Appendix A

**Table A1:** Associations of thrombus burden with ischemic core volume, hypoperfused tissue volume, and penumbra volume in the adjusted analyses.

	Ischemic Core Volume			Hypoperfused Tissue Volume			Penumbra Volume		
	β (95%CI)	R-Squared	*p*-Value	β (95%CI)	R-Squared	*p*-Value	β (95%CI)	R-Squared	*p*-Value
Clot Burden Score	−2.2 (−4.1 to −0.3)	0.33	0.02	−8.7 (−12.8 to −4.5)	0.49	<0.001	−6.3 (−10.2 to −2.4)	0.33	<0.01
Thrombus length	4.7 (−0.1 to 1.1)	0.27	0.13	2.31 (1.1 to 3.6)	0.41	<0.01	1.83 (0.7 to 2.9)	0.27	<0.01
Thrombus location									
ICA	1 (Reference)			1 (Reference)			1 (Reference)		
Proximal M1	−14.1 (−28.9 to 0.6)	0.33	0.06	−16.2 (−45.9 to 13.4)	0.54	0.21	−1.7 (−29.1 to 25.8)	0.40	0.91
Distal M1	−14.6 (−30.1 to 0.9)	0.33	0.06	−27.9 (−58.8 to 3.1)	0.54	0.08	−12.9 (−41.5 to 15.8)	0.40	0.37
M2	−19.3 (−32.9 to −4.8)	0.33	<0.01	−58.6 (−86.3 to −30.1)	0.54	<0.001	−38.8 (−64.4 to −13.1)	0.40	<0.01
M3/M4	−14.3 (−31.3 to 2.7)	0.33	0.09	−80.7 (−115.4 to −45.9)	0.54	<0.001	−65.6 (−97.7 to −33.4)	0.40	<0.001

Adjusted for collateral score, age, sex, and time from stroke onset to baseline imaging.; CI: confidence interval; ICA, internal carotid artery; M1, segment M1 of the middle cerebral artery; M2, segment M2 of the middle cerebral artery; M3, segment M3 of the middle cerebral artery; M4, segment M4 of the middle cerebral artery.

**Table A2:** Associations of thrombus burden with early infarct growth rate, final infarct volume and infarct growth volume in the adjusted analyses.

	Early Infarct Growth rate			Final Infarct Volume			Infarct Growth Volume		
	β (95%CI)	R-Squared	*p*-Value	β (95%CI)	R-Squared	*p*-Value	β (95% CI)	R-Squared	*p*-Value
Clot Burden Score	−1.4 (−3.5 to 0.6)	0.13	0.17	−16.8 (−28.9 to −4.7)	0.27	<0.01	−17.2 (−30.1 to −4.5)	0.24	<0.01
Thrombus length	0.23 (−0.4 to 0.9)	0.18	0.46	1.28 (−3.3 to 5.9)	0.12	0.57	0.97 (−3.5 to 5.4)	0.09	0.66
Thrombus location									
ICA	1 (Reference)			1 (Reference)			1 (Reference)		
Proximal M1	−1.8 (−17.6 to 13.9)	0.19	9.81	−142.8 (−228.9 to −56.7)	0.37	<0.01	−156.2 (−240.3 to −72.1)	0.37	<0.01
Distal M1	−7.8 (−24.3 to 8.9)	0.19	0.35	−114.4 (−205.1 to −23.8)	0.37	0.01	−106.5 (−193.2 to −19.8)	0.37	0.02
M2	−9.1 (−23.4 to 5.5)	0.19	0.22	−172.8 (−259.8 to −85.7)	0.37	<0.001	−166.6 (−251.2 to −82.1)	0.37	<0.001
M3/M4	−5.4 (−23.6 to 12.8)	0.19	0.55	−118.9 (−223.9 to −13.8)	0.37	0.03	−116.3 (−219.7 to −12.8)	0.37	0.03

Adjusted for collateral score, age, and sex. The time from stroke onset to baseline imaging was added to the adjustments for the analyses with final infarct volume and infarct growth volume as outcome measurements. CI: confidence interval; ICA, internal carotid artery; M1, segment M1 of the middle cerebral artery; M2, segment M2 of the middle cerebral artery; M3, segment M3 of the middle cerebral artery; M4, segment M4 of the middle cerebral artery.
